# The Impacts of Social Equity on Health

**DOI:** 10.1089/heq.2021.29009.tge

**Published:** 2021-09-21

**Authors:** Terry Gerton

***Terry Gerton:* Those that are coming behind us have a different level of expectation. They are expecting us to be better and to do better, and they're not going to let us continue the status quo. Welcome to Management Matters, a National Academy of Public Administration podcast where policy meets practice. I'm Terry Gerton, president of the Academy. This month, we're focusing on the issue of social equity. And in this episode, I'll discuss issues surrounding health equity with my guest, Dr. Gail Christopher. Gail is an Academy Fellow, and she's currently the executive director of the National Collaborative for Health Equity and chair of the Trust for America's Health. Gail, thanks so much for joining me today.**

**Dr. Gail Christopher:** Oh, it's my pleasure, Terry.

***Terry Gerton:* Well, I know you have spent your career really as a change agent working on issues around health and well-being and diversity. Certainly, we're seeing that our nation is involved in a very serious conversation over the past year about race and social justice. With all that you've seen and all that you've done, do you think we're in a moment for real racial equity and healing?**

**Dr. Christopher:** I do. You know, I've been at this for a number of decades, and in my lifetime, I've never seen a moment quite like this. We are definitely at a point where there is a critical mass of understanding and support for real transformation. It's not the levels don't hold as high as they did at the pinnacle during the summer months, but there's still a higher level than ever before of willingness to face the challenges that we have as a country and come up with critical decisions and actions. Of course, that's led to more polarization. And so, we have to be very thoughtful and very careful about how we move forward together as a country.

***Terry Gerton:* Well, I know your experience will help inform that conversation. And so, what I want to do now is kind of dig into those areas of expertise that you have because you've been so focused on reducing health inequities. And COVID really has exposed the results of our national approach to health care because of its extraordinary impacts on people and communities of color. So where should we start in developing public policies that can begin to address really systemic health inequity?**

**Dr. Christopher:** That's a good question, Terry. You know, my life I focused on getting us to understand that the bulk of the factors that shape health, that shape the opportunity to be healthy, those factors are within the social domain. The term of art is social determinants of health. It translates into the conditions, in terms of conditions where we live, conditions where we work, conditions where we play, even. Conditions, housing, the air we breathe, the access to transportation, the quality of our lives, that's really what determines health. We, as a country, spend more money on health care, than all of our peer nations. And yet our outcomes are really poor in comparison. There was a wonderful study a few years ago called “Shorter Lives, Poorer Health,” and it compared how we fare as a country with those nations that are our peers economically, right? And clearly, we have to shift our investments from just focusing on the top. If you had a pyramid and you put—it would be like 20% of what determines health is health care. The other 80% have to do with the conditions in which we live. But we spend, on our trillions of dollars that we spend on health in this country, only 3% goes to public health. Three percent goes to affecting those conditions in which people live. And that's what COVID-19 just pull the covers off and made us see that because we don't invest in a strong social infrastructure, an equitable social infrastructure, we have more chronic disease—the data's been there, it's no surprise. But what COVID showed was the increased vulnerability to the infectious diseases as a result of the lifelong exposure and higher incidence of chronic disease. So, this disproportionate impact on communities of color was the result of both excess exposure because of the service level jobs that are predominantly held by people of color, and then the increased exposure to the virus led to a greater viral load in some cases and more incidence of disease, which then accelerated into increased mortality. We can't deny anymore that our system has to change.

***Terry Gerton:* You know, what you just described is a really complicated web of interrelated policies and programs and incentives, both in the social services space and in the health care space. As you've watched over the last 18 months of COVID response, has the urgency of the issue simplified any of that? Are there places where you see people have figured out how to bust all of those silos and really address those core issues?**

**Dr. Christopher:** I'm very proud of this current administration putting equity at the center of its policies. And the COVID relief package that was passed in Congress, really, I think, went to the heart of the matter of the ability to live without fear for eviction, to be able to buy food, to be able to access care, to have some compassion and empathy and sympathy for the crises that we all struggled through that had a disproportionate effect. This administration really embraced all of that, stood in a leadership position, and continues to do so with what I consider all the hallmarks for effective leadership of a democracy, which is pragmatic, empathetic, and compassionate responses to the immediate identified needs. And I'm just very optimistic that you have the wisdom of—you know, some people in the younger groups felt like this incoming administration, our president perhaps was too old, and I think that his experience brought him the wisdom. His experience and his own life crises brought him the capacity to care deeply and to demonstrate that care in ways that resulted in policies that responded. And those policies are, in many ways, the blueprint for the ongoing policies that we will need. We cut child poverty by more than a third, almost in half, but it's a temporary fix. But that should become a permanent fix, because we want children to grow up in environments and within communities where neither them or their parents are experiencing adversity. The data is very clear that childhood adversity leads to more vulnerability to chronic disease later in life. So, addressing the needs of families who have young children, that puts us on par with our peer nations. This is one of those social investments that we had failed to make. But while we're on the subject, I'm going to recommend a book for folks to read that really sort of talks about this. The book is entitled, The Sum of Us, What Racism Costs All of Us and How We Can Prosper Together. Now, in full disclosure my daughter is the author of that book. And even if she were not my daughter though, I would recommend the book. It's a New York Times bestseller and it speaks to the fact that we have to be generous. We have to understand that in a democracy, it's the people that make us viable. And so, we have to be willing to invest in our populations, in our people, so that we maintain our viability not just as a democracy, but also our economic viability.

***Terry Gerton:* Well, so many of those responses that you just articulated were initiated as emergency responses, and we're already seeing some places and function sort of pull back on some of those investments. How does the nation sustain that investment strategy to really institutionalize fundamental change that you're talking about?**

**Dr. Christopher:** You know, I think you just zeroed in on perhaps one of the most important considerations—what does it take to be a leader of a democracy? These decisions to pull back, to cut unemployment benefits, to make these types of decisions, you have leaders of governments and various states making those decisions. And ultimately, how we, as a nation, maintain an infrastructure that is conducive to human development and reduces vulnerability to disease, ultimately, it boils back to who's in charge, who has the power to make the decisions, and do they reflect the sensitivity and the understanding of our needs. I mean, a democracy is a wonderful idea. And that's why these efforts to suppress voting are really scary because the ultimate manifestation, I believe, of our democratic citizenship is our right to vote. People have died to give us that right to vote. Fraud is negligible in terms of the actual incidences of and numbers of. So clearly, these efforts to suppress the vote will interfere with putting leaders in place who are willing to take responsibility, not just for some people, but for the entire population.

***Terry Gerton:* As we continue the conversation about these systemic issues in your new and your current role as the executive director of the National Collaborative for Health Equity, what are the biggest challenges ahead? What are you looking at as we come out of the COVID pandemic that the nation really needs to address?**

**Dr. Christopher:** You know, I think the biggest challenge we face right now is our collective narrative. I think we're in an age of disinformation that is causing us to become more fractured and fractionalized and factionalized as a nation. We can't come out of this hole unless we are whole. And so, we have a challenge to look at this runaway train of social media—this era of artificial intelligence, this era of information overload, disinformation overload. We really do have to find a way to get a handle on what people are hearing, and seeing, and reading, and understanding, so that the messages are helping to foster unity as a country and not division. I don't think we can underestimate the significance of that. In my role as the executive director of the National Collaborative, I understand that the policy decisions that will provide a more equitable playing field to create the conditions for more equitable access to opportunity for health, all of that—all of those, unfortunately, are our public administrative and political decisions. And so being able to have an informed constituency, being able to have people make decisions that demonstrate their own power and their own agency in a realistic and positive way. So, I think one of the biggest challenges we face as a country is bringing these leaders who have benefited so much from this crisis, these artificial intelligence, barons, if you will, bringing them to the table to design appropriate measures for influencing our democracy in a positive way. The other critical challenge that we face is bringing mental health into the center of the conversation. All of us have been affected by this period of unprecedented isolation and separation. It has affected us, not just mentally but physically, in ways that we don't know. Right? And so, bringing that into the forefront of our conversations, we do have a surgeon general now who's written a wonderful book. It's entitled Together, and it talks about how important our social connections are to our well-being. And so, I think this is something we need to lift up in ways that we might not have before.

***Terry Gerton:* You know, I think the comments that you're making about our narrative that the greatest threat to our health is bad information, that mental health is so key to this, are not part of the normal health care narrative. And so, the Biden administration has proposed a significant increase in the public health budget, and a lot of the recovery programs are focused there. But that system has been under incredible strain over the last 18 months. What do we need to do to make sure that public health services consider and address the issues that you're raising and can help us deal with these inequities?**

**Dr. Christopher:** The Robert Wood Johnson Foundation issued a survey result of a poll recently and it revealed how the trust in public health has eroded and how much public health, local public health workers, have been under attack. We've even seen a hollowing out, if you will, of the public health workforce as a result of the politicization of basic human needs and human health needs. So this is a critical time for the public health world. I think we have to step into the chasm as it were. We have to, as public health leaders, we have to be very out front. We have to get people to understand that public health is the backbone of our country. You don't hear about public health when it works. You don't hear about it when we don't have pandemics. You don't hear about it when we have clean water, and healthy air, and safe housing. That's when public health is working, and it gets ignored. And so, again, back to that 3% investment over the decades in public health, we have to change that paradigm. We have to make sure that the nation understands that it is a viable public health system that assures us that we all have the opportunity to be healthy. Yes, we need a viable medical system, but more so, we need the public health infrastructure in place to work. And I hope that every crisis presents an opportunity for growth. I hope that the public health leadership steps into this opportunity.

***Terry Gerton:* We'll see, but it is certainly a national need. And you've been involved in this conversation both from the health care space particularly, but also when you were at the Kellogg Foundation, you were the architect of the effort that they led on “Truth, Racial Healing and Transformation” for America. So, tell us a little bit about that program and what prompted the Kellogg Foundation to initiate it?**

**Dr. Christopher:** Well, I will say that the “Truth, Racial Healing and Transformation,” TRHT, is an adaptation of the globally known and recognized truth and reconciliation process. There was so many—they used the term extrajudicial killings by law enforcement of so many men and boys and women of color, but from my early years at the foundation, I led a precursor to that, which was called “America Healing.” And Terry, I've always seen the intersection between social dislocation, and social inequities, and health inequities. So, when the foundation was committed to being an effective anti-racist organization, and I was vice president for health, it was clear to me that we needed to design a program that brought that together. Over a five-year period, we invested hundreds of millions of dollars to help local communities that were dealing with the issues of racism by bringing communities together, by building bridges. And that was the precursor. We learned lots of lessons. So, as I was approaching retirement, I asked the president and she asked the board if we could, as a foundation, design a truth and reconciliation effort for this country. And that was my last leadership project before retiring from the Kellogg Foundation—to design and implement “Truth, Racial Healing and Transformation.” Now, we say “Truth, Racial Healing, and Transformation,” and not “Truth and Reconciliation,” because we want to be clear that America was conceived and built on the fallacy of a hierarchy of human values. So, it's not about us coming back together, it's about us healing from the harm of that fallacy and transforming our systems that were designed to be inequitable, transforming them into systems that are redesigned now to foster equity, and to do so in a way that connects us and honors our interdependence and our interconnection as a human family. And that's what sets “Truth, Racial Healing and Transformation,” apart from many of the other efforts. Most of the transitional governance efforts or transitional justice efforts have been after a country's been at war, or there's been atrocious leadership by an authoritarian figure and the country is really struggling. We're the only, sort of, mature or seasoned democracy that has centuries, if you will, of this type of division and have never addressed it. And so, in the ways that we have failed to address it in this country—although there are problems all over Europe—but the scale and scope of the problem here, it demands that we be honest about A, our diversity. We have multiple groups in this country and that's another factor. We're looking at entrenched policies and practices, that's another factor. And we also have to recognize that we built ourselves. This country was built on the exploitation and the dehumanization of people based on their physical characteristics. So all of that is built into the framework for TRHT. I'll quickly say, there are five components to the work, to the strategy. One is narrative change; we've got to tell a different story. We've got to be honest. The other is the actual work of healing in communities, bringing diverse people together. I've developed a methodology and approach to that that is informed by what the science tells us about compassion and empathy, and what the neuroscience tells us about creating safe spaces that do not traumatize and create adverse reactions. So, the actual bridgebuilding and healing has to be done. And then we have to address the systemic ways in which racism has been entrenched, and we have three primary pillars of that. One is separation. All kinds of separation, from separating families to separating in terms of residential segregation, transportation, to separation as a primary tool of racial oppression. And then our legal system has been designed to reinforce racial hierarchy, and ultimately, our economy was built on racial hierarchy. So, we have those five pillars of “Truth, Racial Healing and Transformation.” And the good news is, communities are working with this framework across America, doing the work at a grassroots level, and that's very encouraging.

***Terry Gerton:* Well, I know that Congresswoman Barbara Lee has introduced a national referendum that urges the establishment of a United States commission on “Truth Racial Healing and Transformation.” So, given the progress that you're seeing and this recognition of the effort at the congressional level, what do you think is next for this initiative?**

**Dr. Christopher:** You know, I'm so excited that it will continue to grow and expand at the local level. I believe that eventually, we will have a national effort, and Congresswoman Lee is brilliant. She uses the metaphor of the pandemic response, right? She says that we had lots of things happening locally but until we had a national coordinated effort, we definitely were not going to achieve victory. And she thinks that's true for the efforts in terms of racism. It's wonderful that we have so much going on locally, on college campuses across this country right now. The Association of American Colleges and Universities has at least 26 campuses. We think by the end of the summer, there will be more than 50. And then we have several other local jurisdictions. But as an impact in the pandemic response, we need national coordination, and even more so than we did for the pandemic because we're talking about addressing centuries of pathology, if you will. And so, a national coordinated effort is going to be absolutely required. This administration has a lot on its plate right now but I'm hoping that at some point, when things calm down, that the administration will take the lead in establishing a national coordinated effort at “Truth, Racial Healing, and Transformation.”

***Terry Gerton:* Well as we just mentioned, you started this effort while you were at the Kellogg Foundation, and you've probably spent most of your career in the nonprofit sector. What is the role of philanthropy—even if we were to get to a point of a national effort here, what is the role of philanthropy in helping to reduce these kinds of inequities in communications and/or in communities, really, and across the country?**

**Dr. Christopher:** Well, my experience from inside the philanthropic sector is that foundations have the flexibility. They have the flexibility to be catalysts, if you will, for innovation and change. Philanthropy is still committed to “Truth, Racial Healing and Transformation.” Both the Kellogg Foundation and at least 65 or 70 other foundations around the country, mostly local, will still continue to support local efforts. But if we took all the money in all the philanthropy in America, it would be a drop in the ocean as compared to the federal budget and to the federal government. And so, I think the idea of each entity or sector playing its appropriate role, often at the local level for philanthropy, is a catalyst. But, you know, we need to engage public dollars to bring about sustained investment and sustained effort. So, I think it's a combination of both, but I'm really proud of the philanthropic sector because we wouldn't have this moment of racial reckoning if philanthropy had not stepped up. I remember at the Kellogg Foundation, we funded “Black Lives Matter” in its very early stages before it was a recognized Nobel-Prize-winning movement. And we have funded so many of the groups, “The Color of Change” and other groups that have—the civil rights organizations, we had a strategy where we gave operational support to all the civil rights organizations during the last post, the last recession. So, philanthropy has a critical role to play, but it, in and of itself, is catalytic, you need the public sector to be right there as a partner for sustained investment.

***Terry Gerton:* And how do those two sides work best in partnership? How do public administrators and philanthropy first come together to make that kind of institutional change that you're talking about?**

**Dr. Christopher:** I really love that question. You know, the design of TRHT is a local coalition effort. And I can give you one example, for instance, from Illinois. We worked with a local foundation, and they became the hub of the local TRHT effort in Chicago. And as it turned out, the woman who led that foundation was picked by the incoming administration in the state of Illinois to lead health and human services. And one of her efforts was to put out a major RFP in the state of Illinois, to promote racial healing across the state. So ultimately, as you know too well, these things are about relationships. They're about people. They're about people understanding where the opportunity is. Now, they had a relatively modest budget for the local TRHT efforts. They usually have one employee, and they tend to do a lot of networking and building of coalitions strength. But then when a state government issues a major RFP and can put millions behind that, but they are still catalyzing and building on the local efforts. So, I would say the formula is communication and relationship between the sectors. So, the local councils, the philanthropy, the local philanthropic leaders, talking with the leaders of the appropriate agencies in the public sector and figuring out the mechanics of cooperative agreements, of investments, it's one relationship at a time, though, but it's through communication, it's through understanding. We have all these stereotypes. You've got these anti-government folks, right? And then you've got these anti-philanthropic folks. And we just have to realize that those are biases, those are stereotypes. Ultimately, we're all just people trying to make life better, and we have to come together.

***Terry Gerton:* Sounds like it's a lot about getting the right people at the table to have that comprehensive conversation about how to move forward.**

**Dr. Christopher:** It is. And have the common intention. The work of racial healing is about learning how to see in the face of the other, learning how to recognize our interconnectedness and our interdependence as human beings, and letting go of these false taxonomies that divide us. And that is the work of the 21st century. It's time for us to grow up as a human species and realize how much we need one another. This is the opportunity that this moment of reckoning presents for us. I think it's a moment of human development.

***Terry Gerton:* Well, you mentioned at the top and you just come back to this unique moment in the national conversation. I know you articulated that we can all increase our individual and collective capacity for empathy and compassion. And you talk about this collective caring at the core of racial healing. As we wrap up, tell us what you see, and it gives you hope.**

**Dr. Christopher:** I think this generation is their willingness to protest, their willingness to demand an end to injustice, I think that is a beautiful sign that those that are coming behind us have a different level of expectation. They are expecting us to be better and to do better, and they are not going to let us continue the status quo. So that gives me a great deal of hope. Again, the other thing that gives me tremendous hope is the authentic and actual outcome of this past election. We had more voters than we've ever had in the history of our country. People put their lives at risk to vote and to make sure that there was a message for America and from America that said that we're better than this, that we can care about each other, we can grieve the people that are dying, and we can come out of this pandemic a better nation. And so, all of that gives me hope. I happen to be blessed with a grandchild, and I think that is one of life's sweetest gifts. And every time I look at him, I have to have hope for him and for all the children in our country.

***Terry Gerton:* Oh, Gail, I want to just thank you for your lifelong commitment to this issue of equity—an equity in outcomes, and equity in all of our systems and processes, and your continued work in this space. And thanks for spending time with us on the podcast today.**

**Dr. Christopher:** Well, thank you for inviting me and you know, I'm a fan member and supporter of the National Academy of Public Administration, so I was honored to be part of this podcast. Thank you very much, dear.

***Terry Gerton:* Thanks, Gail. For our listeners, check back every Monday for a new podcast from the Academy. We'll be talking to Academy Fellows each week about the challenges facing public administrators at every level of government as we try to make government work and work for all. Thanks for listening.**

